# Individualized Biological Age as a Predictor of Disease: Korean Genome and Epidemiology Study (KoGES) Cohort

**DOI:** 10.3390/jpm12030505

**Published:** 2022-03-21

**Authors:** Seokyung An, Choonghyun Ahn, Sungji Moon, Eun Ji Sim, Sue-Kyung Park

**Affiliations:** 1 Department of Biomedical Science, Graduate School, Seoul National University, Seoul 03080, Korea; seokyung.ann@gmail.com (S.A.); ahnchoonghyun@gmail.com (C.A.); 2 Department of Preventive Medicine, College of Medicine, Seoul National University, Seoul 03080, Korea; kajaman3@snu.ac.kr (S.M.); sim.ej1020@gmail.com (E.J.S.); 3 Cancer Research Institute, College of Medicine, Seoul National University, Seoul 03080, Korea; 4 Interdisciplinary Program in Cancer Biology, College of Medicine, Seoul National University, Seoul 03080, Korea

**Keywords:** biological age, chronic disease, precision medicine, personalized lifestyle medicine, machine learning

## Abstract

Chronological age (CA) predicts health status but its impact on health varies with anthropometry, socioeconomic status (SES), and lifestyle behaviors. Biological age (BA) is, therefore, considered a more precise predictor of health status. We aimed to develop a BA prediction model from self-assessed risk factors and validate it as an indicator for predicting the risk of chronic disease. A total of 101,980 healthy participants from the Korean Genome and Epidemiology Study were included in this study. BA was computed based on body measurements, SES, lifestyle behaviors, and presence of comorbidities using elastic net regression analysis. The effects of BA on diabetes mellitus (DM), hypertension (HT), combination of DM and HT, and chronic kidney disease were analyzed using Cox proportional hazards regression. A younger BA was associated with a lower risk of DM (HR = 0.63, 95% CI: 0.55–0.72), hypertension (HR = 0.74, 95% CI: 0.68–0.81), and combination of DM and HT (HR = 0.65, 95% CI: 0.47–0.91). The largest risk of disease was seen in those with a BA higher than their CA. A consistent association was also observed within the 5-year follow-up. BA, therefore, is an effective tool for detecting high-risk groups and preventing further risk of chronic diseases through individual and population-level interventions.

## 1. Introduction

As the national life span increases, the prevalence of chronic diseases including hypertension (HT), diabetes (DM), and chronic kidney disease (CKD) is accelerating globally [1,2,3]. Chronological age (CA) is a major indicator of health status; however, the effects of CA on diseases may differ based on anthropometry, socioeconomic status (SES), and lifestyle behaviors [4,5,6]. As a result of this difference, people of the same CA have varied biological ages (BA). Therefore, BA, which is calculated using aging markers, has been regarded as a more precise index for assessing health status than CA [7,8,9,10].

Substantial changes in body shape and composition occur with age, and these changes can have an impact on health [6]. In particular, waist circumference is positively associated with the risk of chronic disease [11,12,13]. Differences in aging and health outcomes have also been associated with socioeconomic status [4]. According to the World Health Organization (WHO), chronic diseases share risk factors, including poor lifestyle behaviors such as cigarette smoking, heavy drinking, physical inactivity, and excess body weight [14]. Lifestyle behaviors are also regarded as mediators between SES and health, and they may help alleviate health inequities [15,16,17]. During the coronavirus 2019 (COVID-19) pandemic, health inequities based on socioeconomic disparities have become increasingly pronounced [18]. As a result, the necessity for a personalized health assessment tool is emphasized [19,20,21,22].

There are several previous studies on BA based on clinical information such as laboratory blood tests, frailty-related physical factors, physiological factors, metabolomics, and DNA-methylation [7,23,24,25]. These were useful to understand the biological mechanism of aging, however, were inflexible to control the aging process. Moreover, limited studies were conducted to assess the BA as an index for predicting the risk of disease [26,27]. In this study, BA, which is calculated based on self-assessed risk factors, can be a useful indicator of health status. The combination of these risk factors may relate to increasing BA, which is positively associated with the risk of developing chronic diseases. This suggests that people can regulate the pace of their biological aging by promoting healthy lifestyles and addressing population-level determinants of health.

**Figure 1:**
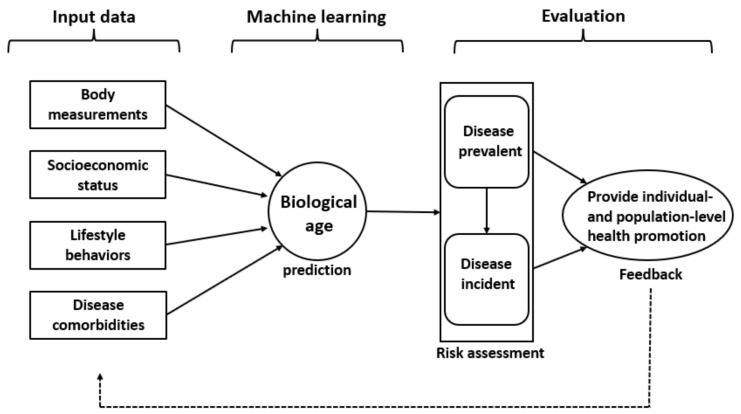
Architecture of development and evaluation of self-assessment of biological age for predicting the risk of chronic disease.

Therefore, this study aimed to develop a personalized BA prediction model based on individual- and population-level risk factors and assess it as a useful indicator for predicting the risk of chronic disease (Figure 1).

## 2. Materials and Methods

### 2.1. Study Population

Participants were drawn from the Korean Genome and Epidemiology Study (KoGES) which integrated three cohorts (including the Ansan and Ansung baseline study from 2001–2002, the health examines study [HEXA] from 2004 to 2013, and the cardiovascular disease association study [CAVAS] from 2005 to 2011). The HEXA study consisted of a total of 173,353 participants aged 40–79 years, which was the largest population dataset from the KoGES. A total of 28,338 individuals aged 40–91 years participated in the CAVAS, and the Ansan and Ansung baseline study consisted of 10,030 individuals aged 40–69 years. All the participants of these three cohorts were interviewed using structured questionnaires and blood tests were collected by well-trained researchers. The detailed design of the KoGES study has been described elsewhere [28]. Of the 211,721 participants in the integrated data, 101,980 healthy individuals with a Charlson’s comorbidity index [29] of 0, who had done body measurements (height, weight, waist, and hip circumference) and completed self-reported information such as SES, comorbidities, and lifestyle behaviors, were included to develop the BA prediction model. To estimate the risk of developing complex chronic disease, 43,143 participants with at least 2 years of follow-up were included (Figure S1).

### 2.2. Biological Age

Among the 128 measurements, variables with missing rates of more than 20% and laboratory data (blood test and calculated dietary intake), which needed to be managed by medical personnel were excluded. Based on previous aging studies, the BA was computed based of the following: (1) body measurement (height, weight, waist, and hip size); (2) SES (income, education level, marital status, and occupation); (3) lifestyle behaviors (smoking duration [years], smoking consumption [packs per day], second-hand smoking [yes/no], drinking frequency [none, 1 time, 2–3 times, 4–6 times/week and daily], frequency of regular exercise [none, 1 time, 2–3 times, 4–6 times/week and daily]); and (4) disease comorbidity (dyslipidemia, asthma, allergy, and thyroid disease).

As there are substantial changes in body shape that occur during the aging process [6], body measurements are useful indicators for estimating biological aging. The relationship between SES and acceleration of aging has been well established [4]. According to previous studies, lifestyle behaviors are also related to chronic diseases and aging [5,14,30,31]. So, lifestyle behaviors were selected as factors affecting BA. Comorbidities were also included. Thus, BA was estimated as a single index using a combination of these self-assessed variables. For women, reproductive factors including age at menarche, oral contraceptive use, and parity were included.

To generate the definite effect of the BA, we defined ‘Age-difference (Age-Diff)’, as the difference between BA and CA (‘Age-Diff’ = BA-CA). The categories of ‘Age-Diff were classified into 4 groups: “Very young BA”, where BA was at least 5-year younger than CA (BA-CA ≤ −5); “Young BA”, where BA was between 1-year and <5-year younger than CA (−5 < BA-CA ≤ −1); “Same BA as CA”, where the BA-CA difference was between −1 year and 1 year (−1 < BA-CA ≤ 1); “Older BA”, where BA was at least 1 year older than CA (BA-CA > 1), respectively.

### 2.3. Outcome Assessments

HT was defined as systolic blood pressure ≥ 130 mmHg or diastolic blood pressure ≥ 80 mmHg or taking any antihypertensive drugs [30]. DM was defined as either a fasting plasma glucose level ≥ 126 mg/dL, HbA1c ≥ 6.5%, or taking any anti-diabetic drugs [31]. CKD was defined as an estimated glomerular filtration rate (eGFR) ≥ 60 mL/min/1.73 m^2^ according to the Chronic Kidney Disease Epidemiology Collaboration (CKD-EPI) [32].

### 2.4. Statistical Analysis

Baseline characteristics were compared according to each chronic condition using a Student’s *t*-test for continuous variables and a chi-squared test for categorical variables. Z-score standardization was performed for continuous elements including body measurements and lifestyle information. Based on the standardized elements, elastic net linear regression [33] was applied to find the optimized coefficients for selected variables that minimize the sum of error squares, which were used to generate our BA prediction model (Figure S2). Our model was then evaluated using 10-fold cross-validation (Figure S3) [34] To estimate the correlation between CA and BA, correlation coefficients (r) were calculated. Logistic regression analyses were performed to calculate the odds ratios (ORs) of chronic diseases according to CA (<50, 50–59, 60–59, and ≥70 years), BA (<50, 50–59, 60–59 and ≥70 years), and Age-Diff (very young BA, young BA, same BA as CA, and older BA). Cox proportional hazards regression analyses were further performed to assess the hazard ratio (HR) of BA and the risk of complex chronic diseases. Further analyses were conducted to estimate the risk of disease within a 5-year follow-up period. All analyses were performed using SAS 9.4 software (SAS Institute, Cary, NC, USA) and R (version 3.3.3) with the ‘glmnet’ package.

## 3. Results

### 3.1. Baseline Characteristics

For BA prediction and to find the association between BA and disease prevalence, a total of 101,980 healthy participants (Charlson’s comorbidity index of ‘0’) aged 40–89 years were included in this study. More than half (65.4%) were women and the mean age at recruitment was 53.0 and 51.9 years for men and women, respectively. Among the total participants, 58,801 had repeat measurements after a median follow-up of 5 (range: 2–13) years. Among the total follow-up subjects, there were new events of DM (2474 subjects), HT (7274 subjects), combination of DM and HT (535 subjects), and CKD (1177 subjects) (Table S1).

### 3.2. Calculation and Assessment of Biological Age

Based on the differences in demographic backgrounds and lifestyle behaviors between sexes, this study developed a sex-specific BA prediction model. BA was calculated based on self-reported questionnaire, including body measurements, SES, comorbidities, and lifestyle behaviors. According to the elastic net regression variable selection process, a total of 20 markers for men and 23 for women were selected. The computed BA was a significantly correlated with CA for men (r = 0.709, *p* < 0.001) and women (r = 0.688, *p* < 0.001), respectively. According to BA predictors, waist circumference, alcohol consumption, and smoking duration were positively associated with BA (Equations (S1)–(S3)).

We found that the oldest CA group (≥70 years) had higher odds of DM (OR: 2.48, 95% CI: 1.93–3.17), HT (OR: 2.66, 95% CI: 2.36–3.00), combination of DM and HT (OR: 3.42, 95% CI: 2.44–4.80), and CKD (OR: 33.42, 95% CI: 26.21–42.61) than those in the youngest CA group (<50 years). The odds of each disease increased more rapidly with an increase in BA. As the BA increased by 1 year, the odds were increased by 6% for DM (OR: 1.06, 95% CI: 1.06–1.07), 7% for HT (OR: 1.07, 95% CI: 1.07–1.08), 10% for combination of DM and HT (OR: 1.10, 95% CI: 1.10–1.11), and 16% for CKD (OR: 1.16, 95% CI: 1.15–1.17). Based on the comparison, BA reflects disease prevalence better than CA. According to the Age-Diff, we found that “Very young BA” group had lower odds of chronic diseases. Compared to the individuals with “Same BA as CA”, lower odds of DM (OR: 0.72, 95% CI: 0.65–0.81), HT (OR: 0.7, 95% CI: 0.68–0.75), combination of DM and HT (OR: 0.65, 95% CI: 0.56–0.76), and CKD (OR: 0.77, 95% CI: 0.65–0.90) were observed in the individuals with “Very young BA” (Table 1)

The risk of developing disease was larger with advanced BA compared to advanced CA. The risk of developing each disease was 1.88-fold for DM (95% CI: 1.28–2.76), 1.57-fold for HT (95% CI: 1.19–2.07), 2.21-fold for combination of DM and HT (95% CI: 0.82–5.99), and 9.62-fold for CKD (95% CI: 6.11–15.14) in the highest CA group, whereas it was 2.68-fold for DM (95% CI: 1.44–5.02), 2.48-fold for HT (95% CI: 1.49–4.11), 5.98-fold for the combination of DM and HT (95% CI: 0.83–43.01), and 13.52-fold for CKD (95% CI: 6.65–27.49) in the “Older BA” group. Compared to the reference group, individuals with “Very young BA” had a decreased risk of DM (HR: 0.63, 95% CI: 0.55–0.72), HT (HR: 0.74, 95% CI: 0.68–0.81), and combination of DM and HT (HR: 0.65, 95% CI: 0.47–0.91). The highest risk of DM (HR: 1.20, 95% CI: 1.07–1.35), HT (HR: 1.15, 95% CI: 1.07–1.23), combination of DM and HT (HR: 1.32, 95% CI: 1.01–1.74), and CKD (HR: 1.20, 95% CI: 0.99–1.45) were observed in the “Older BA” group (Table 2).

A consistent association was also observed for 5 years short-term follow-up period. The “Very young BA” group had a significantly lower risk of DM (HR: 0.66, 95% CI: 0.54–0.80), HT (HR: 0.74, 95% CI: 0.67–0.82), and combination of DM and HT (HR: 0.77, 95% CI: 0.47–1.26) than the reference group (Table 3).

## 4. Discussion

In this study, we developed machine learning-based self-assessment of BA using body measurements, SES, lifestyle factors, and the presence of comorbidity. We found that BA was more strongly associated with the risk of developing chronic diseases than CA. Individuals with a BA lower than the CA have a decreased risk of developing chronic diseases and the risk increased rapidly within a short period of follow-up (within 5 years). A recent study forecasted a continued increase in the global life expectancy [35]. However, the prevalence of comorbidities also increases with age, which decreases quality of life and increases the burden of disease [36,37]. Thus, increasing healthy life expectancy has become more important since the turn of the 21st century. Based on the different health statuses of people of the same CA, several previous studies have come up with BA as an index to represent biological health status. However, most of these studies were based on clinical data such as laboratory blood tests [9,38], physical tests (grip strength and vertical jump) [8], physiological factors (body mass index and percent body fat mass) [9,38], metabolomics [7], and DNA methylation [25]. Although clinical information may reflect the biological aging status, it is difficult for the general population to understand its meaning, and there are restrictions on information collection.

In this study, we computed for BA based on self-assessed variables including body measurement, SES, modifiable lifestyle factors, and the presence of comorbidities. As there are substantial changes in body shape that occur during the aging process [6], body measurements are useful indicators for estimating biological aging. The relationship between SES and acceleration of aging has been well established [4]. According to previous studies, lifestyle modification is effective in decreasing the risk of cardiovascular disease in primary prevention [14,39]. However, there are increasing health inequities due to socioeconomic disparities [18]. Thus, BA might be a valuable predictor of health status, which has an impact on health equity.

Among the risk factors, we found a positive association between waist circumstance and BA. This relationship was supported by prior studies showing that excess body weight is associated with DM, CKD, and cardiovascular diseases [11,12,13]. We also found that lifestyle factors including smoking duration and drinking frequency were significantly associated with BA. This is in line with the J-shaped relationship between alcohol consumption and all-cause and all-cancer mortality in Korea [40]. The causal association with smoking duration also confirmed that smoking increased oxidative stress and inflammation which accelerated aging [41,42,43]. These findings support the idea that lifestyle modification is effective in delaying biological aging. Further research including dietary intake and type of exercise is needed to assess more comprehensive association between the healthy lifestyles and BA.

In this study, we used machine learning algorithms, particularly the elastic net regression to estimate BA. Previous studies have used multiple linear regression and principal component analysis to compute BA, but these methods resulted in overfitting and low interpretability, respectively [44]. Therefore, we selected elastic net linear regression with 10-fold cross-validation to produce a model that minimize overfitting, reduces bias, and are easily to interpret [33,34].

In addition, previous studies on BA have observed the likelihood of diseases by BA in cross-sectional data [8] or the risk of death in cohort data [7,9] rather than observing the risk of developing diseases. In this study, using data from a large cohort of 101,980 Koreans aged 40 to 89 years, we validated that BA could be used as a significant indicator of the risk of developing chronic disease.

One of the strengths of this study is its large sample size. Second, to our knowledge, this is the first approach to advance the study of BA by using factors that are well-measured, well-understood, and easily collected. Because BA consists of modifiable factors, it could be worthwhile to detect high-risk groups and prevent further risks with healthy lifestyle promotion. Third, we could prevent the model overfitting problem by using the elastic net regression model to predict the BA. Finally, we validated that BA is an important indicator of the risk of chronic diseases and their combination in both short (within 5 years) and long follow-up periods.

However, some limitations of this study need to be considered. First, because BA relies on self-reported factors, recall and misclassification biases should be considered. Second, although we examined the combination risk of DM and HT, we could not estimate the risk of when combined with CKD because of the small number of CKD events. Further research is needed to investigate the role of BA in various combinations of chronic diseases. Lastly, due to the lack of data, we were unable to find the association between BA and mortality and frailty. Further study on the effects of BA on mortality and frailty is needed.

## 5. Conclusions

In conclusion, this study suggests that self-assessment of BA could be an effective tool for detecting high-risk groups and reducing disease burden through individual and population-level health promotion.

## 6. Patents

Park, S. (2019). Method and apparatus for generating biometric age prediction model (WO Patent Application No. PCT/KR2018/015516).

## Figures and Tables

**Table 1:** Association with CA, BA, and Age-Diff on the likelihood for the prevalence of DM, HT, combination of DM and HT, and CKD at the baseline among 101,980 cohort participants in the KOGES.

Prevalence at Baseline	Total Cohort N	Chronological Age (CA)		Biological Age (BA) ^1^		Age-Diff (BA-CA) ^2^		
		Cases N	OR (95% CI) ^3^	Cases N	OR (95% CI) ^3^	Age-Diff ^2^	Cases N	OR (95% CI) ^4^
DM								
<50	41,156	915	1.00	854	1.00	Very young BA	759	0.72 (0.65–0.81)
50–59	38,767	1405	1.65 (1.52–1.80)	1970	1.70 (1.57–1.85)	Young BA	788	0.88 (0.79–0.98)
60–69	20,821	1085	2.32 (2.12–2.54)	634	2.76 (2.48–3.06)	Same BA as CA	676	1.00
≥70	1236	72	2.48 (1.93–3.17)	19	2.76 (1.72–4.43)	Older BA	1254	1.17 (1.06–1.29)
Per 1-year increment	101,980	3477	1.04 (1.04–1.05)	3477	1.06 (1.06–1.07)		3477	*p–trend* < 0.001
HT								
<50	41,156	15,977	1.00	14,915	1.00	Very young BA	10,037	0.72 (0.68–0.75)
50–59	38,767	19,873	1.68 (1.63–1.73)	27,683	1.78 (1.73–1.83)	Young BA	10,876	0.89 (0.85–0.92)
60–69	20,821	12,908	2.50 (2.41–2.59)	6777	2.95 (2.81–3.08)	Same BA as CA	9496	1.00
≥70	1236	804	2.66 (2.36–3.00)	187	2.99 (2.27–3.93)	Older BA	19,153	1.24 (1.19–1.29)
Per 1-year increment	101,980	49,562	1.05 (1.04–1.05)	49,562	1.07 (1.07–1.08)		49,562	*p–trend* < 0.001
Combination of DM and HT								
<50	41,156	576	1.00	521	1.00	Very young BA	535	0.65 (0.56–0.76)
50–59	38,767	944	2.13 (1.92–2.37)	1350	2.36 (2.12–2.61)	Young BA	537	0.86 (0.75–0.99)
60–69	20,821	772	3.85 (3.44–4.31)	449	5.12 (4.48–5.85)	Same BA as CA	431	1.00
≥70	1236	40	3.42 (2.44–4.80)	12	5.14 (2.75–9.62)	Older BA	903	1.39 (1.21–1.61)
Per 1-year increment	101,980	2332	1.07 (1.06–1.07)	2332	1.10 (1.10–1.11)		2332	*p–trend* < 0.001
CKD								
<50	41,156	145	1.00	150	1.00	Very young BA	609	0.77 (0.65–0.90)
50–59	38,767	433	3.19 (2.65–3.86)	920	4.61 (3.87–5.48)	Young BA	425	0.98 (0.83–1.16)
60–69	20,821	891	12.49 (10.47–14.90)	512	13.30 (11.07–15.98)	Same BA as CA	235	1.00
≥70	1236	134	33.42 (26.21–42.61)	21	20.91 (12.98–33.68)	Older BA	334	1.51 (1.27–1.80)
Per 1-year increment	101,980	1603	1.15 (1.14–1.16)	1603	1.16 (1.15–1.17)		1603	*p–trend* < 0.001

Abbreviations: CA, chronological age; BA, biological age; DM, diabetes mellitus; HT, hypertension; CKD, chronic kidney disease. ^1^ BA using sex-specific Elastic net model (Supplementary Equations (S1) and (S2)). ^2^ BA-CA difference was classified into four groups: [Very young BA] BA was at least 5-year younger than CA; [Young BA] BA was between 1-year and <5-year younger than CA; [Same BA as CA] BA-CA difference was between −1 year and 1 year; [Older BA] BA was at least 1 year older than CA (>1 year). ^3^ Adjusted for sex. ^4^ Adjusted for sex and chronological age.

**Table 2:** Association with CA, BA, and Age-Diff on the risk of the development of DM, HT, combination of DM and HT, and CKD over total follow-up periods (median 6 years, range 2–13 years) among 101,980 cohort participants in the KOGES.

	Total Cohort N	Chronological Age (CA)		Biological Age (BA) ^1^		Age-Diff (BA-CA) ^2^		
		Cases N	OR (95% CI) ^3^	Cases N	OR (95% CI) ^3^	Age-Diff ^2^	Cases N	OR (95% CI) ^4^
DM								
<50	15,548	735	1.00	698	1.00	Very young BA	491	0.63 (0.55–0.72)
50–59	16,661	1035	1.61 (1.47–1.78)	1429	1.63 (1.49–1.79)	Young BA	591	0.93 (0.83–1.06)
60–69	9127	677	1.81 (1.63–2.01)	337	2.37 (2.08–2.70)	Same BA as CA	473	1.00
≥70	378	27	1.88 (1.28–2.76)	10	2.68 (1.44–5.02)	Older BA	919	1.20 (1.07–1.35)
Per 1-year increment	41,714	2474	1.03 (1.03–1.04)	2474	1.06 (1.05–1.06)		2474	*p–trend* < 0.001
HT								
<50	9977	2822	1.00	2765	1.00	Very young BA	1405	0.74 (0.68–0.81)
50–59	8746	2840	1.38 (1.30–1.45)	3867	1.51 (1.44–1.59)	Young BA	1512	0.86 (0.80–0.93)
60–69	3863	1561	1.73 (1.63–1.84)	627	1.99 (1.82–2.17)	Same BA as CA	1473	1.00
≥70	131	51	1.57 (1.19–2.07)	15	2.48 (1.49–4.11)	Older BA	2884	1.15 (1.07–1.23)
Per 1-year increment	22,717	7274	1.03 (1.02–1.03)	7274	1.05 (1.04–1.05)		7274	*p–trend* < 0.001
Combinationof DM and HT								
<50	7107	193	1.00	183	1.00	Very young BA	1047	0.65 (0.47–0.91)
50–59	5796	208	1.63 (1.32–2.02)	303	1.95 (1.59–2.38)	Young BA	135	1.10 (0.82–1.46)
60–69	2250	130	2.36 (1.84–3.03)	48	3.03 (2.15–4.27)	Same BA as CA	100	1.00
≥70	77	4	2.21 (0.82–5.99)	1	5.98 (0.83–43.01)	Older BA	196	1.32 (1.01–1.74)
Per 1-year increment	15,230	535	1.05 (1.03–1.06)	535	1.07 (1.06–1.09)		535	*p–trend* < 0.001
CKD								
<50	15,808	200	1.00	222	1.00	Very young BA	450	0.86 (0.71–1.03)
50–59	17,069	359	2.39 (2.01–2.85)	722	2.83 (2.43–3.29)	Young BA	258	0.81 (0.67–0.98)
60–69	9215	597	7.36 (6.26–8.64)	225	5.82 (4.83–7.02)	Same BA as CA	191	1.00
≥70	368	21	9.62 (6.11–15.14)	8	13.52 (6.65–27.49)	Older BA	278	1.20 (0.99–1.45)
Per 1-year increment	42,460	1177	1.11 (1.10–1.12)	1177	1.12 (1.11–1.13)		1177	*p–trend* < 0.001

Abbreviations: CA, chronological age; BA, biological age; DM, diabetes mellitus; HT, hypertension; CKD, chronic kidney disease. ^1^ BA using sex-specific Elastic net model (Supplementary Equations (S1) and (S2)). ^2^ BA-CA difference was classified into four groups: [Very young BA] BA was at least 5-year younger than CA; [Young BA] BA was between 1-year and <5-year younger than CA; [Same BA as CA] BA-CA difference was between −1 year and 1 year; [Older BA] BA was at least 1 year older than CA (>1 year). ^3^ Adjusted for sex. ^4^ Adjusted for sex and chronological age.

**Table 3:** Association with CA, BA, and Age-Diff on the risk of the development of DM, HT, combination of DM and HT, and CKD on short-term follow-up periods (≤5 years) among 101,980 cohort participants in the KOGES.

	Total Cohort N	Chronological Age (CA)		Biological Age (BA) ^1^		Age-Diff (BA-CA) ^2^		
		Cases N	OR (95% CI) ^3^	Cases N	OR (95% CI) ^3^	Age-Diff ^2^	Cases N	OR (95% CI) ^4^
DM								
<50	15,548	292	1.00	295	1.00	Very young BA	251	0.66 (0.54–0.80)
50–59	16,661	514	1.67 (1.45–1.93)	692	1.72(1.50–1.97)	Young BA	310	1.02 (0.86–1.22)
60–69	9127	373	2.18 (1.87–2.54)	202	2.81 (2.35–3.37)	Same BA as CA	219	1.00
≥70	378	18	2.43 (1.51–3.92)	8	3.76 (1.86–7.61)	Older BA	417	1.32 (1.11–1.56)
Per 1-year increment	41,714	1197	1.04 (1.03–1.05)	1197	1.06 (1.05–1.06)		1197	*p–trend* < 0.001
HT								
<50	9977	1586	1.00	1516	1.00	Very young BA	966	0.74 (0.67–0.82)
50–59	8746	1909	1.38 (1.29–1.48)	2667	1.66 (1.56–1.77)	Young BA	980	0.84 (0.77–0.92)
60–69	3863	1140	1.86 (1.73–2.01)	478	2.19 (1.97–2.43)	Same BA as CA	931	1.00
≥70	131	36	1.68 (1.21–2.35)	10	2.26 (1.21–4.22)	Older BA	1794	1.21 (1.11–1.32)
Per 1-year increment	22,717	4671	1.03 (1.03–1.04)	4671	1.05 (1.05–1.06)		4671	*p–trend* < 0.001
Combinationof DM and HT								
<50	7107	87	1.00	80	1.00	Very young BA	217	0.77 (0.47–1.26)
50–59	5796	106	1.87 (1.33–2.63)	166	2.29 (1.66–3.17)	Young BA	115	1.23 (0.80–1.91)
60–69	2250	79	3.18 (2.17–4.64)	29	3.83 (2.37–6.20)	Same BA as CA	78	1.00
≥70	77	4	5.67 (2.04–15.77)	1	9.63 (1.32–70.28)	Older BA	115	1.47 (1.10–1.98)
Per 1-year increment	15,230	276	1.06 (1.04–1.08)	276	1.08 (1.06–1.11)		525	*p–trend* = 0.002
CKD								
<50	15,808	62	1.00	70	1.00	Very young BA	62	0.88 (0.66–1.16)
50–59	17,069	151	2.33 (1.73–3.13)	330	3.63 (2.81–4.70)	Young BA	72	0.83 (0.62–1.11)
60–69	9215	299	3.91 (6.94–12.01)	117	7.70 (5.72–10.37)	Same BA as CA	49	1.00
≥70	368	13	10.46 (5.74–19.04)	8	24.41 (1.68–51.04)	Older BA	93	1.44 (0.93–2.23)
Per 1-year increment	42,460	525	1.13 (1.12–1.14)	525	1.13 (1.12–1.15)		276	*p–trend* = 0.049

Abbreviations: CA, chronological age; BA, biological age; DM, diabetes mellitus; HT, hypertension; CKD, chronic kidney disease. ^1^ BA using sex-specific Elastic net model (Supplementary Equations (S1) and (S2)). ^2^ BA-CA difference was classified into four groups: [Very young BA] BA was at least 5-year younger than CA; [Young BA] BA was between 1-year and <5-year younger than CA; [Same BA as CA] BA-CA difference was between −1 year and 1 year; [Older BA] BA was at least 1 year older than CA (>1 year). ^3^ Adjusted for sex. ^4^ Adjusted for sex and chronological age.

## Data Availability

Raw data were generated from the Korea Genome and Epidemiology Study (KOGES). The derived data supporting the findings of this study are available from the corresponding author upon request.

## Supplementary Materials

The following supporting information can be downloaded at: https://www.mdpi.com/article/10.3390/jpm12030505/s1, Table S1: Baseline characteristics of healthy participants at baseline among 103,912 cohort participants in the Korean Genome and Epidemiology Study (KOGES), Figure S1: Selection algorithm of study subjects for the analyses among 101,980 cohort participants in the Korean Genome and Epidemiology Study (KOGES), Figure S2: Coefficient paths for the Elastic Net model (A). Mean-Squared Error (B), Figure S3: Diagram of 10-fold cross-validation, Equation (S1): Equation of biological age in men, Equation (S2): Equation of biological age in women, Equation (S3): An example of biological age.
