# Supplementary material for: Individualized Biological Age as a Predictor of Disease: Korean Genome and Epidemiology Study (KoGES) Cohort
Source: J Pers Med. 2022 Mar 21;12(3):505. doi: 10.3390/jpm12030505 (PMC8955355; doi:10.3390/jpm12030505)
Supplement: Supplementary file 1 [file jpm-12-00505-s001.zip › jpm-1607629-supplementary.pdf]

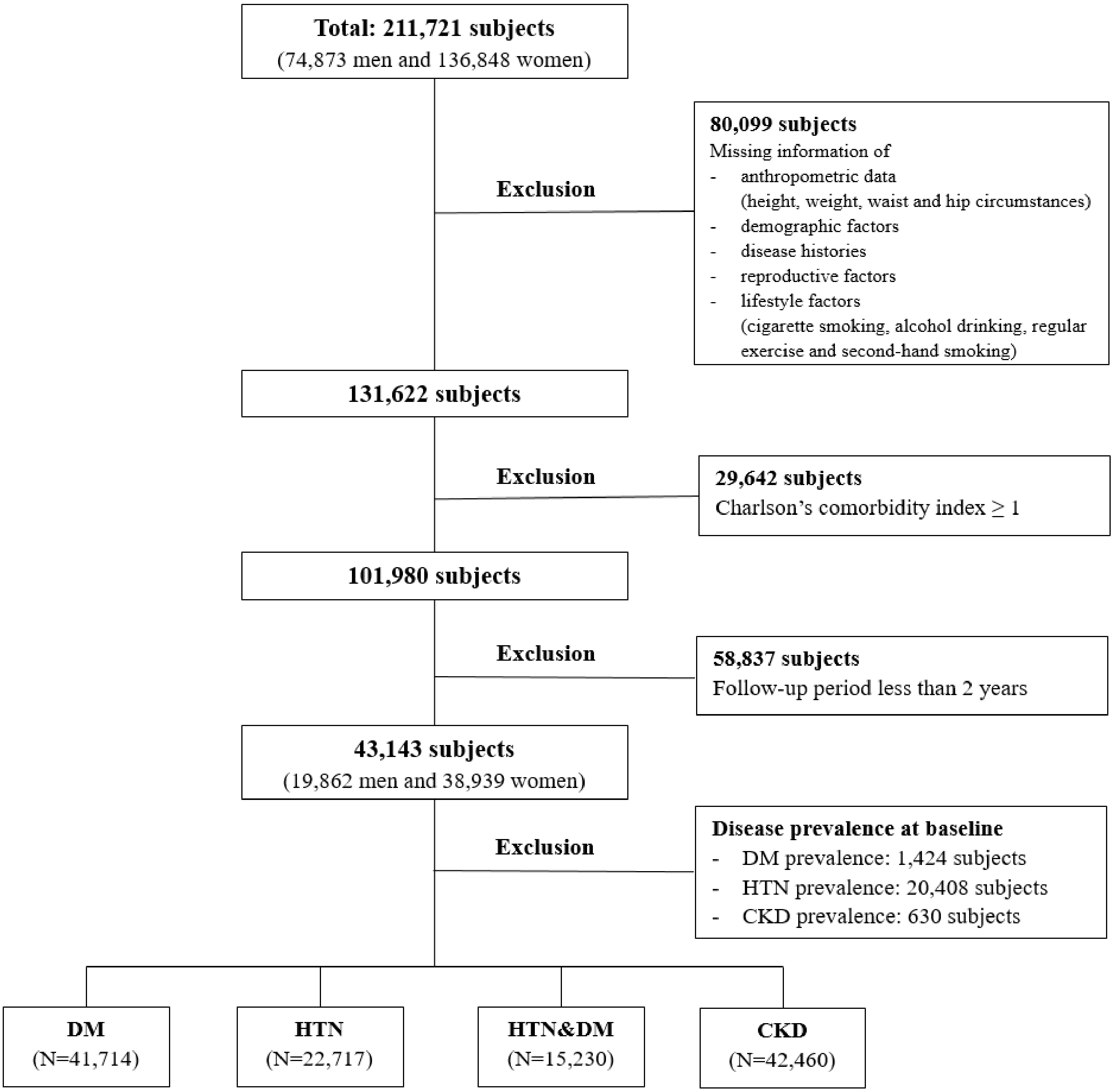

Figure S1. Selection algorithm of study subjects for the analyses among 101,980 cohort participants in the Korean Genome and Epidemiology Study (KOGES).

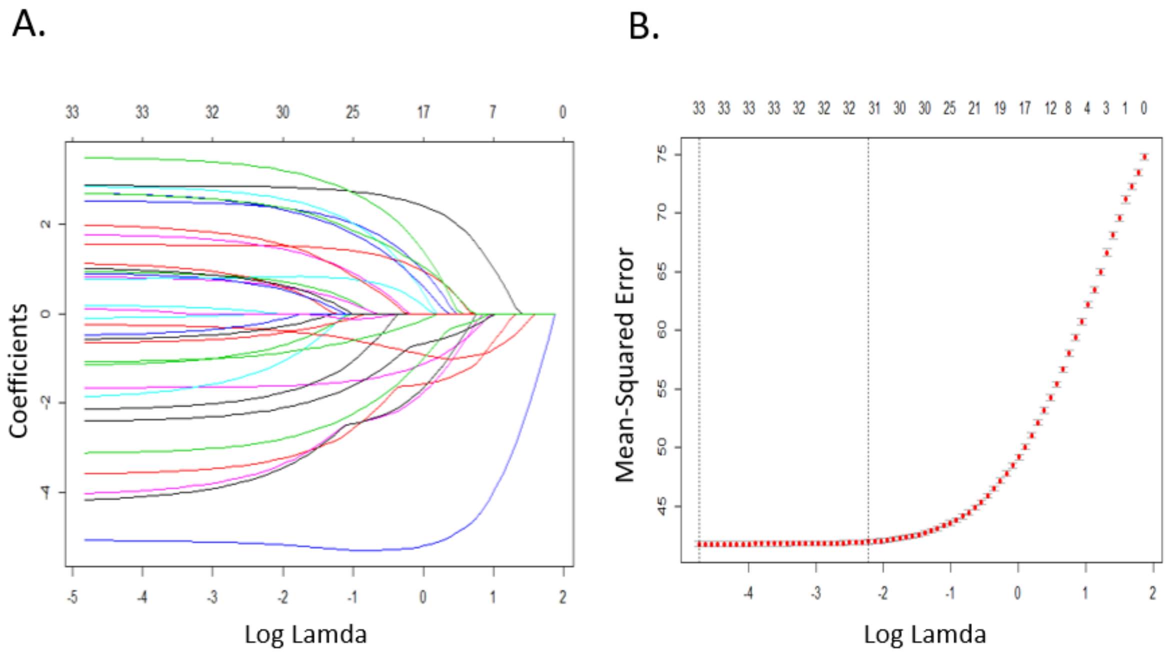

Figure S2. Coefficient paths for the Elastic Net model (A). Mean-Squared Error (B).

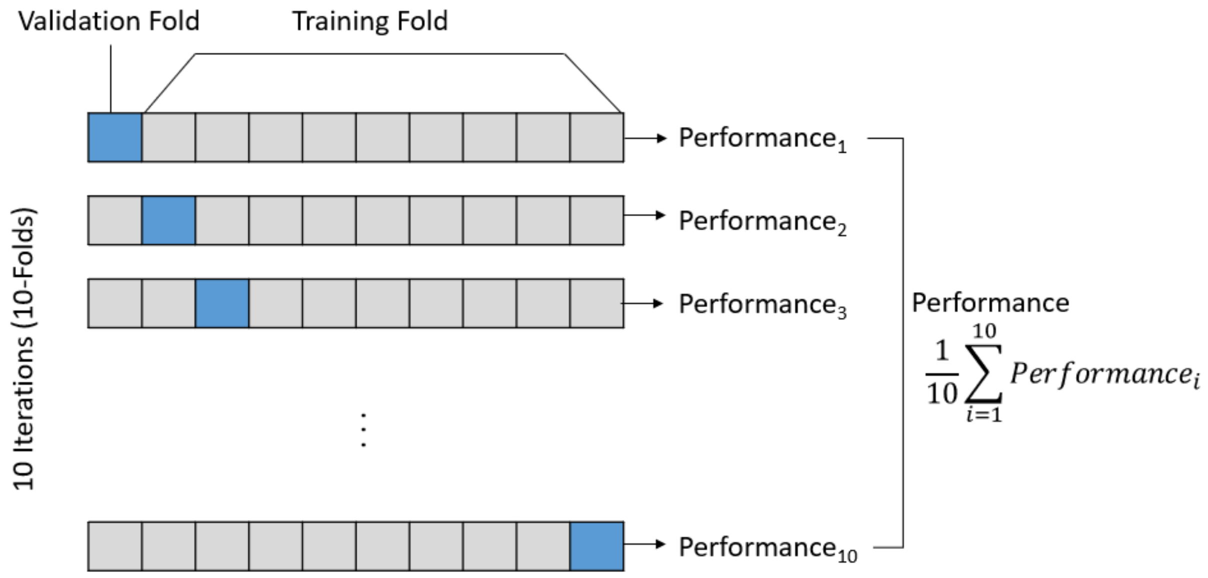

Figure S3. Diagram of 10-fold cross-validation

Table S1. Baseline characteristics of healthy participants at the baseline among 103,912 cohort participants in the Korean Genome and Epidemiology Study (KOGES)

**Variables**	**Cohort participants with CCI=0 at baseline (n=101,980)**		**Non-diabetes cohort participants at baseline (n=41,714)***	
	**Men (n=35,331)**	**Women (n=66,649)**	**Men (n=13,693)**	**Women (n=28,021)**
	**Mean ± SD**	**Mean ± SD**	**Mean ± SD**	**Mean ± SD**
Age, years	53.0 ± 8.58	51.9 ± 7.97	53.9 ± 8.41	52.1 ± 7.78
Height, cm	168.7 ± 5.84	156.3 ± 5.43	168.6 ± 5.78	156.3 ± 5.38
Weight, kg	69.5 ± 9.33	57.9 ±7.74	69.4 ± 9.01	57.8 ± 7.63
Waist circumstance, cm	85.5 ± 7.50	78.5 ± 8.29	85.3 ± 7.33	78.3 ± 8.31
Hip circumstances, cm	95.7 ± 5.69	93.5 ± 5.75	95.7 ± 5.53	93.3 ± 5.65
	**No. (%)**	**No. (%)**	**No. (%)**	**No. (%)**
College or more	12,673 (35.9)	12,939 (19.4)	5,115 (37.4)	5,501 (19.6)
Have occupation	30,281 (85.7)	29,523 (44.3)	11,334 (82.8)	11,667 (41.6)
Income ≥ $4,000	9,471 (26.8)	15,120 (22.7)	3,634 (26.5)	6,437 (23.0)
Current smokers	11,801 (33.4)	1,496 (2.3)	3,885 (28.4)	438 (1.6)
Current drinkers	26,321 (74.5)	22,299 (33.5)	10,114 (73.9)	8,788 (31.4)
Regular exercise	18,928 (53.6)	32,295 (48.5)	7,825 (57.2)	14,456 (51.6)
	**Non-hypertension cohort participants at baseline (n=22,717)***		**Non-CKD cohort participants at baseline (n=42,460)***	
	**Men (n=5,733)**	**Women (n=16,984)**	**Men (n=14,025)**	**Women (n=28,435)**
Age, years	53.5 ± 8.35	50.7 ± 7.50	53.8 ± 8.36	52.1 ± 7.75
Height, cm	168.7 ± 5.75	156.7 ± 5.30	168.6 ± 5.76	156.3 ± 5.38
Weight, kg	67.9 ± 8.76	56.9 ± 7.24	69.5 ± 9.08	57.9 ± 7.69
Waist circumstance, cm	84.0 ± 7.29	76.9 ± 7.89	85.4 ± 7.37	78.4 ± 8.38
Hip circumstances, cm	95.0 ± 5.49	92.8 ± 5.49	95.8 ± 5.57	93.4 ± 5.67
	**No. (%)**	**No. (%)**	**No. (%)**	**No. (%)**
College or more	2,282 (39.8)	3,934 (23.2)	5,222 (37.2)	5,553 (19.5)
Have occupation	4,808 (83.9)	7,306 (43.0)	11,645 (83.0)	11,891 (41.8)
Income ≥ $4,000	1,616 (28.2)	4,532 (26.7)	3,749 (26.7)	6,519 (22.9)
Current smokers	1,779 (31.0)	308 (1.8)	4,030 (28.7)	460 (1.6)
Current drinkers	4,000 (69.8)	5,483 (32.3)	10,413 (74.3)	8,896 (31.3)
Regular exercise	3,260 (56.9)	8,810 (51.9)	7,997 (57.0)	14,669 (51.6)

CCI, Charlson comorbidity index; CKD, Chronic kidney disease; *Participants with at least 2 years of follow-up.

Supplementary Equation S1. Equation of biological age in men

$$[\textbf{Biological Age in Men}] = 65.1 + 0.6\left(\frac{[Age(year)]-53.0}{8.57}\right) - 0.4\left(\frac{[Height(cm)]-168.7}{5.83}\right) - 2.6\left(\frac{[Weight(Kg]-69.5}{9.32}\right) + 2.1\left(\frac{[Waist(cm)]-85.5}{7.48}\right) - 0.03\left(\frac{[Hip(cm)]-95.7}{5.68}\right) + 0.10\,[(Dyslidemia)\text{Yes} = 1; \text{No} = 0] - 0.6\,[(Allergy]; \text{Yes} = 1; \text{No} = 0] + 1.3\,[(Thyroid\ disease);\ \text{Yes} = 1; \text{No} = 0] + 0.8\,[(Asthma);\ \text{Yes} = 1; \text{No} = 0] - 5.6\,[(\text{Smoking status});\ \text{None} = 0;\ \text{Past} = 1;\ \text{Current} = 0] - 10.1\,[(\text{Smoking status});\ \text{None} = 0;\ \text{Past} = 0;\ \text{Current} = 1] + 4.7\left(\frac{Smoking\ duration(Year)-17.3}{14.14}\right) - 0.6\left(\frac{Cigarette\ per\ day-12.3}{11.21}\right) + 0.2\,[(Drinking\ status); \text{None} = 0; \text{Past} = 1; \text{Current} = 0] - 1.0\,[(Drinking\ status); \text{None} = 0; \text{Past} = 0; \text{Current} = 1) - 1.3\,[(Secondhand\ smoking); \text{Yes} = 1; \text{No} = 0] + 1.1\,[(Regular\ exercise); \text{Yes} = 1; \text{No} = 0] - 1.9\,[(Income\ level); < \$1{,}000 = 0; \$1{,}000 - \$2{,}000 = 1; \$2{,}000 - \$4{,}000 = 0; \geq \$4{,}000 = 0;] - 4.0\,[(Income\ level); < \$1{,}000 = 0; \$1{,}000 - \$2{,}000 = 0; \$2{,}000 - \$4{,}000 = 1; \geq \$4{,}000 = 0;] - 4.2\,[(Income\ level); < \$1{,}000 = 0; \$1{,}000 - \$2{,}000 = 0; \$2{,}000 - \$4{,}000 = 0; \geq \$4{,}000 = 1;] - 2.2[(Education); < M\text{iddle school or less} = 0; \text{High school} = 1; \text{College or more} = 0] - 3.5[(Education); \text{Middle school or less} = 0; \text{High school} = 0; \text{College or more} = 1] + 3.4[(Marital\ status); \text{Single} = 0; \text{Married} = 1] - 4.8[(Have\ occupation); \text{Yes} = 1; \text{No} = 0]$$

*Standardization was done for continuous elements based on the study population

Supplementary Equation S2. Equation of biological age in women

$[\textbf{Biological Age in Women}] = 57.3 + 1.107\left(\frac{[Age(year)]-51.9}{7.96}\right) - 0.8\left(\frac{[Height(cm)]-56.3}{5.42}\right) - 1.1\left(\frac{[Weight(Kg]-57.9}{7.73}\right) + 2.0\left(\frac{[Waist(cm)]-78.5}{8.28}\right) - 1.0\left(\frac{[Hip(cm)]-93.5}{5.74}\right) + 2.6\,[(Dyslidemia)\text{Yes} = 1; \text{No} = 0] - 0.7\,[(Allergy]; \text{Yes} = 1; \text{No} = 0] + 0.7\,[(Thyroid\ disease);\ \text{Yes} = 1; \text{No} = 0] + 0.7\,[(Asthma);\ \text{Yes} = 1; \text{No} = 0] - 2.1\,[(\text{Smoking status});\ \text{None} = 0;\ \text{Past} = 1;\ \text{Current} = 0] - 3.4\,[(\text{Smoking status});\ \text{None} = 0;\ \text{Past} = 0;\ \text{Current} = 1] + 0.5\left(\frac{Smoking\ duration(Year)-0.5}{3.18}\right) - 0.2\left(\frac{Cigarette\ per\ day-0.3}{2.15}\right) - 1.7\,[(Drinking\ status); \text{None} = 0; \text{Past} = 1; \text{Current} = 0] - 2.2\,[(Drinking\ status); \text{None} = 0; \text{Past} = 0; \text{Current} = 1) - 0.8\,[(Secondhand\ smoking); \text{Yes} = 1; \text{No} = 0] + 1.0\,[(Regular\ exercise); \text{Yes} = 1; \text{No} = 0] - 2.1\,[(Income\ level); < \$1{,}000 = 0; \$1{,}000 - \$2{,}000 = 1; \$2{,}000 - \$4{,}000 = 0; \geq \$4{,}000 = 0;] - 3.7\,[(Income\ level); < \$1{,}000 = 0; \$1{,}000 - \$2{,}000 = 0; \$2{,}000 - \$4{,}000 = 1; \geq \$4{,}000 = 0;] - 3.9\,[(Income\ level); < \$1{,}000 = 0; \$1{,}000 - \$2{,}000 = 0; \$2{,}000 - \$4{,}000 = 0; \geq \$4{,}000 = 1;] - 3.2[(Education); \text{Middle school or less} = 0; \text{High school} = 1; \text{College or more} = 0] - 4.5[(Education); \text{Middle school or less} = 0; \text{High school} = 0; \text{College or more} = 1] - 1.8[(Marital\ status); \text{Single} = 0; \text{Married} = 1] - 1.9[(Have\ occupation); \text{Yes} = 1; \text{No} = 0] + 1.4\,(\frac{Age\ at\ menarche(Year)-15.2}{1.84})$ +0.9[(Have history of oral contraceptive); Yes=1; No=0] + 2.5[(Have history of pregnant); Yes=1; No=0]

*Standardization was done for continuous elements based on the study population

Supplementary Equation S3. An example of biological age

Consider a 50-year-old married man with non-smoking, current drinking habit, secondhand smoke, without regular exercise, has an occupation with income level over 200 - 400K/KW, graduated with college, height of 170 cm, weight of 68.5 kg, waist size of 85 cm, hip size of 96 cm and without any of disease history in 2005.

$$[\textbf{Biological Age in Men}] = 65.1 + 0.6\left(\frac{[50(year)]-53.0}{8.57}\right) - 0.4\left(\frac{[170(cm)]-168.7}{5.83}\right) - 2.6\left(\frac{[68.5(Kg)]-69.5}{9.32}\right) + 2.101\left(\frac{[85(cm)]-85.5}{7.48}\right) - 0.03\left(\frac{[96(cm)]-95.7}{5.68}\right) + 0.1\,[(Dyslidemia)\text{Yes} = 1; \text{No} = 0] - 0.6\,[(Allergy]; \text{Yes} = 1; \text{No} = 0] + 1.3\,[(Thyroid\ disease);\ \text{Yes} = 1; \text{No} = 0] + 0.817\,[(Asthma);\ \text{Yes} = 1; \text{No} = 0] - 5.6\,[(\text{Smoking status});\ \text{None} = 0;\ \text{Past} = 1;\ \text{Current} = 0] - 10.1\,[(\text{Smoking status});\ \text{None} = 0;\ \text{Past} = 0;\ \text{Current} = 1] + 4.7\left(\frac{0(Year)-17.3}{14.14}\right) - 0.6\left(\frac{0-12.3}{11.21}\right) + 0.2\,[(Drinking\ status); \text{None} = 0; \text{Past} = 1; \text{Current} = 0] - 1.0\,[(Drinking\ status); \text{None} = 0; \text{Past} = 0; \text{Current} = 1) - 1.3\,[(Secondhand\ smoking); \text{Yes} = 1; \text{No} = 0] + 1.1\,[(Regular\ exercise); \text{Yes} = 1; \text{No} = 0] - 1.9\,[(Income\ level); < \$1{,}000\ = 0; \$1{,}000 - \$2{,}000 = 1; \$2{,}000 - \$4{,}000 = 0; \geq \$4{,}000 = 0;\,] - 4.0\,[(Income\ level); < \$1{,}000\ = 0; \$1{,}000 - \$2{,}000 = 0; \$2{,}000 - \$4{,}000 = 1; \geq \$4{,}000 = 0;\,] - 4.2\,[(Income\ level); < \$1{,}000\ = 0; \$1{,}000 - \$2{,}000 = 0; \$2{,}000 - \$4{,}000 = 0; \geq \$4{,}000 = 1;\,] - 2.2[(Education); \text{Middle school or less} = 0; \text{High school} = 1; \text{College or more} = 0] - 3.5[(Education); \text{Middle school or less} = 0; \text{High school} = 0; \text{College or more} = 1] + 3.4[(Marital\ status); \text{Single} = 0; \text{Married} = 1] - 4.8[(Have\ occupation); \text{Yes} = 1; \text{No} = 0]$$

∴ Biological age = 47.8
